# Clinical and functional properties of novel *VHL* mutation (X214L) consistent with Type 2A phenotype and low risk of renal cell carcinoma

**DOI:** 10.1111/j.1399-0004.2010.01464.x

**Published:** 2011-06

**Authors:** AD Sorrell, S Lee, C Stolle, J Ellenhorn, A Grix, WG Kaelin, JN Weitzel

**Affiliations:** a City of Hope National Medical CenterDuarte, California; b Dana-Farber Cancer Research InstituteBoston, Massachusetts; c The Children's Hospital of PhiladelphiaPhiladelphia, Pennsylvania; d The Permanente Medical GroupSacramento, California; e Howard Hughes Medical InstituteChevy Chase, Maryland, USA

**Keywords:** germline mutation, HIF, Jun B, neuroendocrine, pheochromocytoma, von Hippel Lindau

## Abstract

Sorrell AD, Lee S, Stolle C, Ellenhorn J, Grix A, Kaelin Jr WG, Weitzel JN. Clinical and functional properties of novel *VHL* mutation (X214L) consistent with Type 2A phenotype and low risk of renal cell carcinoma.

This report describes clinical characteristics in families with a Type 2A phenotype and functional properties of a novel von Hippel Lindau variant (X214L). Pedigrees were analyzed. Analysis of von Hippel Lindau (*VHL*) coding exons and flanking intronic sequences in DNA from a proband with pheochromocytoma and islet cell tumor was performed. Western blot assays for VHL protein (pVHL), HIF*α*, and Jun B were conducted using *VHL* null renal clear carcinoma cell lines that were engineered to produce wild-type or X214L mutant pVHL. Pedigree analysis indicated that the variant tracked with disease and the same or similar *VHL* point mutations were identified in several Type 2A families. The predicted 14 amino acid extended pVHL variant, when reintroduced into *VHL* null cells, was stable and retained the ability to downregulate HIF*α* in a hydroxylation-dependent manner. In contrast, the variant was defective with respect to downregulation of JunB. pVHL X214L, like other pVHL variants associated with a low risk of clear cell renal carcinoma, largely preserves the ability to downregulate HIF. In contrast, this variant, like other pVHL variants linked to Type 2A disease, fails to suppress JunB. This underscores that JunB may play a role in the pathogenesis of Type 2A *VHL* disease.

## Introduction

Von Hippel Lindau (*VHL*) syndrome is a rare autosomal dominant disorder that causes an array of potentially disabling and life-threatening tumors in children and adults. Deleterious mutations in the *VHL* gene cause tumors such as hemangioblastomas (HB) of the retina, cerebellum, brainstem, and spine; clear renal cell carcinomas (RCCs); pheochromocytomas (Ph); neuroendocrine tumors (N); endolymphatic sac tumors (ELST); cystadenomas of the epididymus, broad ligament, and pancreas; and cysts in the kidneys and pancreas 1,2). RCC affects approximately 40% of *VHL* patients, and metastatic RCC is the most common cause of death in *VHL* germline carriers 3–5. *VHL* disease penetrance is age dependent with clinical symptoms developing in 19% of affected individuals by 15 years of age, 52% by age 25, and 91% by the age of 45 (6). The age-related penetrance and the specific types of tumors that develop in affected individuals depend upon the functional consequences of the specific *VHL* germline mutation.

**Fig 1:**
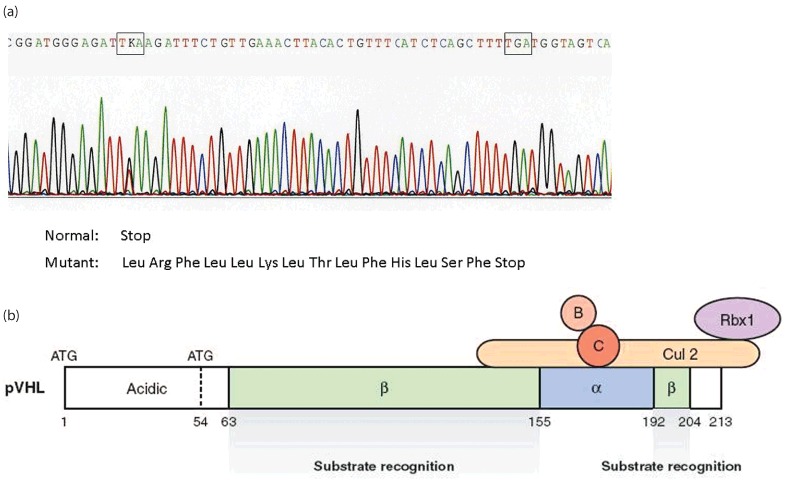
(a) DNA sequence analysis of the mutant *VHL* gene. The normal stop codon (TGA) is changed to a codon for leucine (TTA) at amino acid position 214, leading to an extension peptide containing 14 novel amino acids and a new stop codon. The original stop codon and the stop codon of the extension peptide are boxed. K, heterozygous for a G and a T at the same position in the DNA sequence. A portion of the translated amino acid sequence indicating the normal stop codon and the sequence of the extended peptide is shown below the nucleotide sequence. (b) Illustration of the 213 amino acids comprising the full-length VHL protein (pVHL). pVHL contains alpha (*α*) and beta (*β*) subdomains. The *α* subdomain binds to elongin C (C), Cullin 2 (Cul2), elongin B (B), and Rbx1. The beta subdomain is important for recognition and binding to substrates such as hypoxia-inducible factor 1 alpha. (modified from Ref (9)).

The *VHL* tumor suppressor gene is comprised of three exons (639 nt), located on the short arm of chromosome 3 (3p26-p25; OMIM 608537). The *VHL* gene encodes for a protein comprised of 213 amino acids (24.2 kDa) and contains two protein binding domains, the beta domain (comprised of residues 63–155 and 193–204) and the alpha domain (residues 155–193) (Fig. 1). The VHL protein (pVHL) utilizes the alpha domain to connect to Elongin B, Elongin C, and CUL2 to form a functional VHL complex, whereas the beta domain is used to recognize substrates (7). The VHL complex regulates the expression of at least 17 cellular proteins, including a key transcription factor known as the hypoxia-inducible factor 1 (HIF). Overabundance of HIF permits the overproduction of growth factors, which stimulate the oncogenic processes that lead to both hereditary and sporadic forms of clear RCC 8,9.

We report the clinical characteristics and functional consequences of a novel *VHL*, missense, mutation (X214L). Analysis of this mutation supported the Type 2A phenotype in families with a previously unclassifiable molecular subtype of *VHL* disease.

## Materials and methods

### Patient selection

The proband was referred to the City of Hope (COH) by her surgical oncologist. The proband and her mother were enrolled in a COH IRB-approved Hereditary Cancer Biology Research (HCBR) registry after informed consent. Three additional probands and four at risk familial germline carriers with the same or similar genotypes were identified through a database search by the Molecular Genetics Laboratory at the Children's Hospital of Philadelphia (MGL-CHOP). Additional phenotype data was solicited from the five physicians who referred these patients to the MGL-CHOP, consistent with current HIPAA regulations. One physician responded, his patient subsequently provided informed consent and enrolled in the HCBR registry. Medical records and pathologic data were available for the COH cases and one MGL-CHOP case.

### DNA analysis

Boston University School of Medicine's laboratory performed CLIA-approved DNA full sequence analysis of the *VHL* gene for the proband and site-specific DNA analysis for the proband's mother. Genomic DNA was extracted from peripheral blood lymphocytes using standard procedures. Sequence analysis consisted of polymerase chain reaction (PCR) amplification and automated fluorescence sequencing of all three *VHL* exons. Multiple ligation probe amplification (MLPA), using the *VHL* kit from MRC-Holland was also performed. The *VHL* kit from MRC-Holland detects approximately 97% of all deletion mutations in the *VHL* gene.

### Functional characterization

786-O and RCC-4 *VHL* null RCCs were cultured in Dulbecco's modified medium supplemented with 10% fetal bovine serum and 1% penicillin/streptomycin. X214L mutant cDNA plasmid was generated from a wild-type pVHL construct by site-directed mutagenesis. A reading frame for 14 extra amino acids was inserted with primers that introduced a 5′BamHI site and a 3′EcoRI site, followed by subcloning into a pBabe retroviral vector that introduces an N-terminal hemagglutinin (HA)-epitope tag. RCCs were infected with retroviruses and selected in 2 µg/ml puromycin for 7–10 days.

### Western blot analysis

Twenty micrograms of whole cell extract per lane, measured by Bradford assay, was resolved on 10% or 12% SDS-PAGE gels and transferred to nitrocellulose membrane (Bio-Rad) to detect endogenous HIF2*α* and JunB. After blocking in TBS with 5% nonfat milk, the membranes were probed with anti-HIF2*α* monoclonal antibody (UP-11; raised in our laboratory) (10) or anti-JunB monoclonal antibody (C-11; Santa Cruz Biotechnology) or monoclonal anti-HA antibody (HA-11; Santa Cruz). Bound protein was detected with horseradish peroxidase (HRP)-conjugated secondary antibodies and an enhanced chemiluminescence kit (Pierce).

## Results

### Phenotype

**Fig 2:**
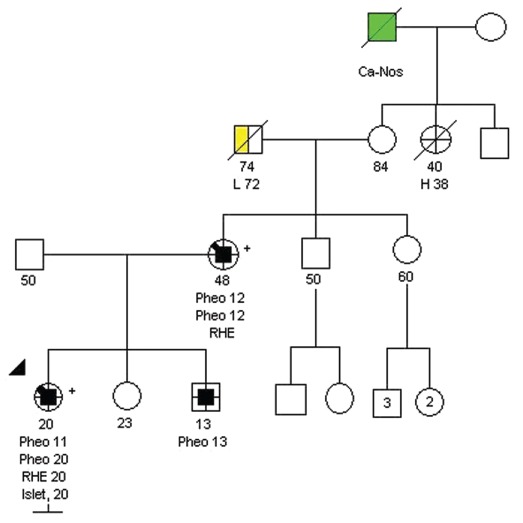
Pedigree of family with *VHL* X214L germline mutation. The index patient (proband) is indicated by 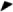. Family members with confirmed *VHL* gene mutations are identified by the plus sign (+). The square symbols represent males; the circle symbols represent females. The age (years) of each individual is noted below the symbol. Family members affected by cancer are identified by diagnoses abbreviations and symbols [pheochromocytoma (Pheo 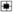), liver cancer (L 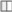), cancer not otherwise specified (Ca–Nos 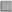), Hodgkin's (H 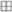), islet cell tumor of the pancreas (Islet), retinal hemangioblastoma (RHE 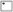)]. Diagonally dashed symbols represent deceased individuals.

Our proband is a 20-year-old female referred for cancer genetics consultation due to a personal history of two synchronous primary tumors (a second primary pheochromocytoma and a malignant neuroendocrine tumor of the pancreas) and a family history of early-onset pheochromocytomas. A four-generation pedigree was obtained (Fig. 2).

She presented with a less than 6-month history of non-specific abdominal pain, nausea, vomiting, diarrhea, fatigue, and 10–15 pounds of intentional weight loss. Biochemical screening demonstrated an increased normetanephrine level of 487 (normal less than 111); plasma metanephrine level was normal at 47. A diagnostic computed tomography (CT) scan of the abdomen revealed a 3.7 × 4.1 cm ring enhancing mass in the head of the pancreas and an additional mass in the right adrenal gland. No pancreatic or renal cysts were identified. Pathological review confirmed the presence of a 1.6 cm islet cell neuroendocrine carcinoma of the pancreas with nodal metastasis (4 out of 14 lymph nodes were positive) and a benign pheochromocytoma (2 cm in size) associated with medullary hyperplasia of the right adrenal gland. Screening for *VHL*-associated tumors revealed bilateral retinal HB. Since her surgery 20 months ago, our proband has shown no evidence of recurrent disease.

### Genotype

Full sequence analysis on our proband and site-specific testing of our proband's mother identified a point mutation (X214L) in exon three of the *VHL* gene (Fig. 1). The X214L (c.641G>T; p.X214LeuextX15) mutation replaces the normal stop codon with a leucine. This run-on mutation, which is predicted to increase the length of the normal pVHL by 14 amino acids, had not been reported in the literature and the clinical significance was unknown.

The MGL-CHOP identified stop codon mutations in three other patients with *VHL* disease. The MGL-CHOP patient who participated in HCBR registry carries a heterozygous X214C VHL germline mutation (c.642A>T; p.X214CysextX15). This A to T mutation at nucleotide 642 of the VHL gene replaces the stop codon (TGA) in exon 3 with a codon for cysteine (TGT), which is predicted to cause the same elongated protein product seen in our proband and two other MGL-CHOP patients. The specific mutations identified in the other two patients are as follows: X214L (c.641G>T; p.X214LeuextX15) and X214T (c.642 A>G; p.X214TrpextX15).

The three MGL-CHOP patients who carry stop codon mutations express similar phenotype characteristics as those seen in our X214L family (Table 1). Patient X214C is a 62-year-old male with a history of bilateral pheochromocytomas, multiple central nervous system (CNS) and spinal HB, retinal angiomas, and the synchronous intraoperative finding of a clear RCC with a neuroendocrine tumor of the pancreas. He has undergone bilateral adrenalectomies and several surgical procedures for his spinal tumors. Patient X214L is a 38-year-old female with bilateral retinal angiomas. Patient X214T is a 32-year-old female with pheochromocytoma and pancreatic cysts. The age of onset of disease and family histories are not available for these patients.

**Table 1:** Genotype–phenotype characteristics of *VHL* (X214L) and similar point mutations

				Phenotype	
VHL mutation	Amino acid (aa) substitution^a^	Hydropathy index^b^	Molecular weight (aa)	Tumor-types	Age at diagnosis (years)
X214L	Leucine	+3.8	131	Bilateral pheochromocytoma	Proband – 11^d^, 20^d^ Proband's mother – 12
				Malignant pancreatic neuroendocrine^c^	Proband – 20
two cases (female)				Bilateral retinal hemangioblastomas	Proband – 20^d^
					Mother – 40^d^
X214L one case (female)	Leucine	+3.8	131	Bilateral retinal hemangioblastomas	Unknown (gene tested at age 38)
X214C one case (male)	Cysteine	+2.5	121	Clear cell renal carcinoma^c^ and pancreatic neuroendocrine^c^	62
				Bilateral pheochromocytomas	
				Multiple CNS and spinal hemangioblastomas	Unknown
				Retinal hemangioblastomas	
X214T one case (female)	Tryptophan	−0.7	204	Pheochromocytoma pancreatic cysts	Unknown

VHL, von Hippel Lindau.

a Standard amino acid side chain properties are similar for each substitution (i.e. nonpolar, neutral charge at pH 7.4, and hydrophobic).

b Number represents the hydrophobic (+) or hydrophilic (−) properties of the amino acid side chain (21). Amino acids with higher numbers are more hydrophobic and tend to be located on the internal surface of three dimensional protein structure models (22). Hydrophilic amino acids tend to be found on protein surfaces.

c Synchronous.

d Screen-detected tumor.

### Functional data

**Fig 3:**
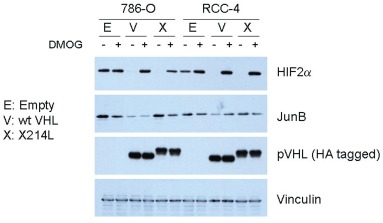
Immunoblot analysis using two VHL protein (pVHL) null renal carcinoma cell lines (786-O and RCC-4). Where indicated, cells were treated with 1mM of dimethyloxaloylglycine (DMOG), dissolved in dimethyl sulfoxide (DMSO), for 16 h to induce HIF2*α* protein. DMOG-induced HIF2*α* protein in the X214L mutant (X) and wt VHL (V) expressing cells. Compared to wt VHL, high-levels of JunB expression was seen in the X214L mutant expressing cell lines, pre- and post-DMOG exposure. No pre- and post-DMOG expression differences were detected in the cells expressing the empty vector (E). This data represents three independent experiments.

Prior studies suggest that pVHL mutants linked to familial pheochromocytoma fail to downregulate the c-Jun antagonist JunB, attenuating apoptosis, and setting the stage for neoplastic transformation (10). Using two independent, pVHL null, RCC lines (786-O and RCC-4), we found that the X214L pVHL mutant is associated with abnormal JunB expression, when compared to wild-type pVHL (Fig. 3). The electrophoretic mobility of the exogenous HA-tagged pVHL (X214L) was slower than that of HA-tagged wild-type pVHL, consistent with the presence of 14 additional amino acids. HIF2*α* induction patterns were assessed after treatment with dimethyloxaloylglycine (DMOG), a prolyl hydroxylase inhibitor, in cell lines expressing wild-type or X214L pVHL. The X214L mutant pVHL appeared to downregulate HIF*α* protein expression, in a canonical, hydroxylation-dependent, manner. In contrast, X214L was defective relative to wild-type pVHL with respect to downregulation of JunB. In sum, X214L mutant protein was associated with low HIF2*α* expression and high JunB expression, a pattern consistent with a low risk of kidney cancer and high risk of pheochromocytoma.

## Discussion

This report provides clinical and functional data for a novel *VHL* variant of uncertain significance (X214L) that is now classifiable as a deleterious mutation. The functional work included in this report supports prior studies that show Jun B dysregulation plays a role in the pathogenesis of Type 2A *VHL* disease (10).

Phenotype expression of *VHL* is highly variable with marked genotype–phenotype correlation 11–14. *VHL* Type 2 families have a high risk of developing pheochromocytoma. The majority of *VHL* Type 2 mutations occur in the Elongin C binding area (EloC 157-170), which cause only a partial loss of *VHL* functional ability (15). Type 2 *VHL* families are divided into subgroups (Types 2A, 2B, and 2C), based on their risk for HB, clear RCC, and characteristics of their germline mutation. Families with Type 2A disease typically carry the more functional ‘surface’ type of missense mutations. Surface missense mutations are associated with the highest rates of age-related risk for pheochromocytoma (∼60% penetrance by age 60). Type 2A missense mutations are associated with a high risk of HB, but they are associated with a low risk of RCC (16). Interestingly, although Type 2C patients also carry missense types of *VHL* germline mutations, they do not have an increased risk of developing either HB or RCC. *VHL* Type 2B families are more similar to Type 1 families, in that they carry more functionally deleterious mutations (partial gene deletions, nonsense mutations, and ‘deep’ missense mutations) that cause a high risk of renal cancer and a high risk of HB. ‘Deep’ missense mutations cause more structural damage, because they tend to be hydrophobic residues buried deep within the protein structure (17). Despite the seemingly hydrophobic nature of the *VHL* X214L missense mutation, this variant has maintained the ability to regulate HIF expression, which is predictive of a low RCC risk. Additional studies are required (e.g. crystal structure of protein, quantitative functional investigations, etc.) to elucidate the unique biological features of this variant.

Pancreatic islet cell tumors are a rare component of the *VHL* phenotype that has been reported in both Type 2A and Type 2B patients. Less then 12 % of *VHL* patients express both neuroendocrine tumors of the pancreas and pheochromocytomas (1). Pediatric *VHL* germline carriers, almost, never express this combination of tumors (18). We contribute an additional case of bilateral pheochromocytoma combined with early-onset neuroendocrine tumor of the pancreas to the literature. The phenotype in this proband and family support findings from a large case series reported by Walther et al (19) who found a statistically significant association (p < 0.025) between mutations at nucleotides 382 through 482 of the *VHL* gene and the early-onset of pheochromocytomas.

This stop codon mutation predicts the translation of a ‘run-on’ protein, extending the length of the normal pVHL by 14 amino acids. Similar mutations that create elongated protein products are causative in other inherited disorders. For instance, single base substitutions in the alpha-chain termination codon produce elongated alpha-chain hemoglobin variants that cause an alpha-thalassemia phenotype (20). We identified four families with pheochromocytoma-dominant *VHL* phenotypes who carry stop codon mutations. Although the elongated pVHL length is the same, the amino acid encoded by the stop codon mutation is different in three of the four families, making shared family origin very unlikely. A search of the *VHL* mutational databases at Boston University's School of Medicine and the Worldwide *VHL* Mutations Database revealed the existence of a French family (15) with a germline mutation in the same codon. However, no clinical information (published or anecdotal) about this family could be obtained.

This report expands the knowledge of genotype–phenotype correlations in *VHL* and reinforces the need to perform functional investigations of variants of uncertain significance in disorders with strong phenotype–genotype correlations.
